# A Review of Artificial Intelligence in Embedded Systems

**DOI:** 10.3390/mi14050897

**Published:** 2023-04-22

**Authors:** Zhaoyun Zhang, Jingpeng Li

**Affiliations:** 1 School of Electrical Engineering and Intelligentization, Dongguan University of Technology, Dongguan 523000, China; 2 School of Computer Science and Technology, Dongguan University of Technology, Dongguan 523000, China; klee305@126.com

**Keywords:** artificial intelligence, embedded intelligence, model compression, resource-constrained device, hardware accelerator, internet of things

## Abstract

Advancements in artificial intelligence algorithms and models, along with embedded device support, have resulted in the issue of high energy consumption and poor compatibility when deploying artificial intelligence models and networks on embedded devices becoming solvable. In response to these problems, this paper introduces three aspects of methods and applications for deploying artificial intelligence technologies on embedded devices, including artificial intelligence algorithms and models on resource-constrained hardware, acceleration methods for embedded devices, neural network compression, and current application models of embedded AI. This paper compares relevant literature, highlights the strengths and weaknesses, and concludes with future directions for embedded AI and a summary of the article.

## 1. Introduction

Over the years, the development of artificial intelligence and its applications has greatly reduced the complexity of many machine learning models, making it easier to deploy them on resource-constrained devices. Furthermore, corresponding support for models and algorithms on these devices has emerged. These developments have facilitated a new research direction: embedded artificial intelligence [1,2,3]. The concept of embedded AI was first introduced in reference [3], which proposed that the IoT could evolve into the Wisdom Web of Things (W2T) and emphasized that embedded intelligence about individuals, the environment, and society could increase the number of users of existing IoT systems, promote environmental sustainability, and enhance social awareness. Recent developments in embedded AI are described in references [1,2], both of which combine embedded AI with IoT technology. The current mainstream research direction of embedded AI is to integrate it with IoT, which includes edge computing with convolutional accelerator [4] and load distribution [5]. Reference [6] mentions that the combination of embedded intelligence and IoT is the future direction of development. In addition, edge computing can be combined with artificial intelligence, resulting in what is known as edge intelligence [7].

The current development of embedded AI is two-way: the optimization of AI models and algorithms reduces the difficulty of deploying them on embedded devices, while hardware accelerators in embedded devices increase support for AI models and algorithms. Furthermore, hardware resources are being developed, and AI is rapidly advancing in mobile devices. For example, reference [8] describes the deployment of neural networks on cell phones, and there are also neural networks specifically designed for mobile devices, such as MobileNet [9]. MobileNet will be discussed in detail in Section 3 on lightweight networks.

To deploy artificial intelligence in embedded devices, several aspects need to be considered: (1) choosing a suitable deployment platform, (2) using hardware accelerators, as mentioned in the second section, (3) applying model compression techniques, as described in the third section [10,11], and (4) enhancing hardware support for AI algorithms and models. All these points are discussed in detail in this paper.

In this paper, we will introduce embedded AI from the following aspects: the first section introduces the development history of embedded AI, the second section introduces the hardware accelerators of embedded AI, the third section introduces the key technologies of embedded AI, the fourth section introduces the application directions of embedded AI, the fifth section discusses the challenges encountered in the current development of embedded AI and provides an outlook for the future, and finally the sixth section concludes with a summary.

## 2. Hardware Acceleration Methods for Embedded AI

The current acceleration of embedded AI can be achieved through three types of hardware: FPGA, ASIC, and GPU. FPGA (Field-Programmable Gate Array) is a type of programmable logic chip; ASIC (Application-Specific Integrated Circuit) is an integrated circuit specifically designed to meet the needs of a particular application; and a GPU (Graphics Processing Unit) is a processor used for processing graphics data.

### 2.1. FPGA

FPGA (Field Programmable Gate Array), also known as Field Programmable Gate Arrays, can generate high-performance, low-power hardware mappings in neural networks due to its flexibility. FPGA can be customized to meet system-level requirements in different environments, such as throughput, latency, and power.

The authors of paper [12] propose an approach for accelerating convolutional neural networks on FPGAs to generate high-performance hardware designs by assembling pre-implemented components of convolutional neural networks into a graph topology-based puzzle. The use of pre-implemented components allows minimal resource utilization for predicting performance, which reduces engineering time and increases yields. The pre-implemented components in the paper are implemented using an out-of-context design approach. Experiments show a 69% yield improvement compared to traditional FPGA implementations, achieving more than 1.5 times the maximum frequency with fewer resources and higher energy efficiency. Reference [13] proposes the ASimOV framework, which consists of two parts: (1) an AI simulator for finding the optimal parameters in the parameter search space of the AI accelerator and algorithm to achieve maximum performance (e.g., precision); and (2) an HDL code generator for using the optimal parameters of the AI accelerator and algorithm to generate a hardware model, which is implemented in the FPGA for functional testing purposes. This end-to-end framework allows for the optimization of the AI accelerator on specific datasets, while also reducing actual cost. The functionality of ASimOV has been verified in small AI algorithms such as character recognition, clothing recognition, and speech recognition. For the k-NN AI accelerator, the total performance reached 90.7% with 64kb of memory. Reference [14] compares artificial neural networks and spiking neural networks on FPGAs in terms of method, learning process, and efficiency using the MNIST, GTSRB, and CIFAR-10 datasets. Spiking neural networks were found to be slightly faster than artificial neural networks for the learning process when using the MNIST dataset. In terms of power consumption, spiking neural networks consume less FPGA logic and have 19% more power than artificial neural networks. The authors compared the spiking neural network with the convolutional neural network on the GTSRB dataset and found that the former saved 42% of power, but the high latency of the inference process ultimately did not result in energy savings. On the CIFAR-10 dataset, the test results showed that overall FPGA logic decreased, with a 75% reduction in logic conversion efficiency, resulting in energy savings compared to the GTSRB dataset mentioned above because the inference time was greatly reduced. The increasing complexity of convolutional neural network models has made it challenging to map them to FPGA devices. Therefore, fpgaConvNet research was conducted to reduce the difficulty of deploying network models. For instance, in reference [15], the authors propose that the inference process of ConvNet could be interpreted as a streaming application. fpgaConvNet uses a computational synchronous data flow model as its modeling core, where synchronous data flow is mainly used in the design and analysis of parallel systems. By transforming the design space exploration task into a multi-objective optimization problem, fpgaConvNet can effectively target applications with various performance requirements, either in terms of high throughput or low latency. The authors present experiments comparing fpgaConvNet with a cluster of embedded GPUs. fpgaConvNet generates a more energy-efficient accelerator and achieves higher raw performance within the same power constraints compared to highly optimized embedded GPU designs. The authors argue that fpgaConvNet deploys the ConvNet model on embedded FPGAs, which provides the necessary infrastructure. Reference [16] proposes an end-to-end optimized framework called fpgaConvNet, which is an automated mapping framework for convolutional networks on FPGAs, and an automated design approach for the synchronous dataflow paradigm while defining a set of SDF transformations. A system multi-objective optimization formulation is also proposed for fpgaConvNet, enabling it to generate co-optimized hardware designs for convolutional neural network workloads, target devices, and application performance metrics. In the experiments, the authors compare the performance of fpgaConvNet with the existing FPGA-related work. The comprehensive experimental structure demonstrates that fpgaConvNet achieves outstanding results in terms of performance, performance density, and other relevant metrics.

There are two approaches to deploying algorithms on FPGA: enhancing the algorithm to leverage the hardware capabilities of FPGA, or optimizing the execution efficiency of the algorithm in hardware. Most current work focuses on memory compression to maximize operational efficiency, but does not consider energy consumption on the FPGA platform. Additionally, it is important to consider the operational efficiency on the FPGA when selecting the programming language.

### 2.2. ASIC

ASIC (Application Specific Integrated Circuit) is a type of special-purpose integrated circuit that is highly customized and less programmable, and therefore relies heavily on algorithm design. To deploy neural networks on ASICs, appropriate hardware accelerators need to be designed for the specific network structure. The optimization of hardware accelerators involves utilizing parallel resources and reducing computational complexity.

Reference [17], on the other hand, proposes an optimization approach that utilizes parallel resources. The authors introduce the YodaNN convolutional neural network accelerator, which simplifies the design complexity by using simpler complementary operations and multiplexers instead of fixed-point MAC units. Experimental results demonstrate that the YodaNN accelerator achieved 2.7 times higher peak performance and 32 times higher energy efficiency than the state-of-the-art CNN accelerator at that time. Reference [18] proposes the UCNN (Unique Weight CNN Accelerator), which is a convolutional neural network accelerator optimized for reducing the computational effort. As the convolutional neural network design evolves, the size and number of filters in the network structure will keep increasing, and the values of the weights are bound to be repeated. Therefore, each common weight can be extracted from the multiplication it involves; for example, the $w_{i}$ in the following equation can be extracted.

(1) $$\begin{array}{l} b=w_{i}\times a_{i}+w_{i}\times a_{j}+w_{i}\times a_{k}\\ b=w_{i}\times (a_{i}+a_{j}+a_{k}) \end{array}$$

In addition, the author proposes maintaining two hardware structures: the input indirect table and the weight indirect table. F1 and F2 in the figure below are two filters, x and z are weights, and IF1 is the feature map, which represents the convolution between the computational filter and the feature map. The operation in F1 can be approximately replaced by the operation in F2, thus reducing the computational effort.

(2) $$\begin{array}{l} F1:x*(c+g+a+h)+z*(b+d+f+e+k)\\ F2:x*(c+g)+z*(a+h)+x*(b+d+f)+z*(e+k) \end{array}$$

Reference [19] proposes MSICs (ML-specific integrated circuits), ASICs dedicated to deep neural networks. These circuits use larger on-chip buffers instead of off-chip ones and exploit reusability and sparsity to achieve higher energy efficiency by reducing numerical precision and optimizing data flow. Experimental results show that this deep learning-specific ASIC is over 10 times more energy efficient than a CPU or GPU.

The main approach for deploying networks on ASIC is to create neural network accelerators. Networks can be deployed on ASIC through quantization, which can be further optimized by combining both quantization and neural network accelerator techniques. Additionally, a hardware accelerator can be improved by utilizing sparse architectures.

### 2.3. GPU

GPU (graphics processing unit) is a specialized processor used for accelerating the speed of image processing in hardware. Currently, GPUs are also widely used to accelerate neural networks in machine learning. In this paper, we mainly introduce the role of GPUs in enhancing the security of machine learning.

Reference [20] proposes CRYPTGPU, a privacy-preserving machine learning system that uses a cryptographic interface and a “GPU-friendly” cryptographic protocol to maintain privacy when performing both linear and nonlinear operations on the GPU. Experimental results show that the GPU-private convolution protocol is more than 150 times faster than similar CPU-based protocols, and about 10 times faster than CPU in simulations for nonlinear operations such as ReLU activation. It is concluded that GPUs can accelerate private training and inference for deep learning, and enable privacy-preserving deep learning on complex datasets and networks. Another paper, [21], proposes ParSecureML, a GPU-based framework that improves the performance of secure machine learning algorithms based on two-party computation in a secure context. It addresses problems of complex computation patterns, frequent intra-node data transfers between CPUs and GPUs, and complex inter-node data dependencies by implementing profiling-guided adaptive GPU utilization, fine-grained double pipeline intra-node CPU-GPU collaboration, and compressed transmission intra-node communication. Experimental results show that ParSecureML improves acceleration by an average of 33.8× over state-of-the-art secure machine learning frameworks, with an average acceleration of 31.7× when applied to inference.

The above two papers address security in machine learning, which is an undeveloped field of research. However, it may be a promising direction for embedded AI in the future.

### 2.4. Other Acceleration Hardware

In addition to the above three platforms, reference [22] deploys a pedestrian image detection system on a neuromorphic chip and compares it to a CPU system and a GPU system, demonstrating improved efficiency and reduced energy consumption compared to traditional GPU-accelerated and CPU multi-core systems. This approach is particularly beneficial for resource-constrained platforms. The authors also suggest areas for improvement, noting that the neuromorphic chip NM500 used in the paper supports only one hidden layer, which may limit the functionality of deep neural networks, and lacks dynamic switching capability, making it difficult to manage power consumption. Finally, they suggest that integrating the neuromorphic chip into System on Chips (SOC) could improve the performance and energy efficiency of machine learning on embedded systems. In another paper [23], the authors optimize and deploy a deep neural network on the NVIDIA Jetson platform, which supports more parallel computing capabilities and multiple output interfaces compared to other platforms. This facilitates the deployment of artificial intelligence algorithms and models on the Jetson platform.

### 2.5. Summary

The various acceleration methods for the three hardware platforms mentioned in 2.1, 2.2, and 2.3 have their own advantages in embedded AI applications and are suitable for different scenarios. They should be chosen according to different needs. If the scenario requires the frequent switching of computational tasks, FPGA can provide higher flexibility and programmability. If the scenario requires deep learning tasks with parallel computation on large amounts of data, GPUs can provide higher computational performance. If the applications have fixed computational tasks, ASICs can provide higher performance and energy efficiency. Of course, these three platforms can also work together. For example, in [24], the authors combined these two hardware platforms by exploiting the high-density computational performance of GPUs in machine learning and the low latency demonstrated by FPGAs in model inference. Table 1 provides the characteristics of the various approaches for each deployment platform in this chapter.

## 3. The Key Technologies of Embedded AI

To deploy neural networks on resource-constrained devices such as embedded systems, several problems need to be addressed. Firstly, how can embedded devices carry huge models and algorithms of artificial intelligence with their limited resources? Secondly, how can hardware devices support algorithms and models after meeting the above conditions? This chapter proposes three key techniques to solve these challenges. Section 3.1 and Section 3.2 address model compression and algorithm optimization to meet the hardware constraints of resource-constrained devices, while Section 3.3 focuses on algorithm optimization on hardware to enhance support for AI algorithms. Model compression aims to maintain model accuracy while reducing its size as much as possible, while binarization networks aim to compress the model to the smallest possible size while improving model accuracy. Additionally, the chapter explores CPU/GPU algorithms by investigating the support provided by CPUs and GPUs for artificial intelligence algorithms.

### 3.1. Model Compression of Neural Network

#### 3.1.1. Network Structure Redesign

The network structure design is a method of improving existing neural networks by designing new network structures. Many researchers have undertaken significant work in this area.

**Figure 1:**
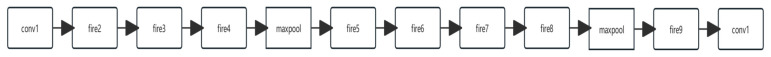
SqueezeNet network structure.

In 2017, Landola et al. [25] proposed SqueezeNet, a lightweight network that maintains accuracy using fewer parameters. SqueezeNet consists of two parts: a convolutional neural network architecture designed by the authors, and the Fire module. To maintain accuracy, three strategies were used in designing the convolutional neural network architecture: 1. using 1 × 1 filters instead of partial 3 × 3 filters, 2. using a squeeze layer to reduce the input channels of 3 × 3 filters, and 3. delaying downsampling (postponing the downsampling process to the end of the network). The network structure of SqueezeNet is shown in Figure 1.

**Figure 2:**
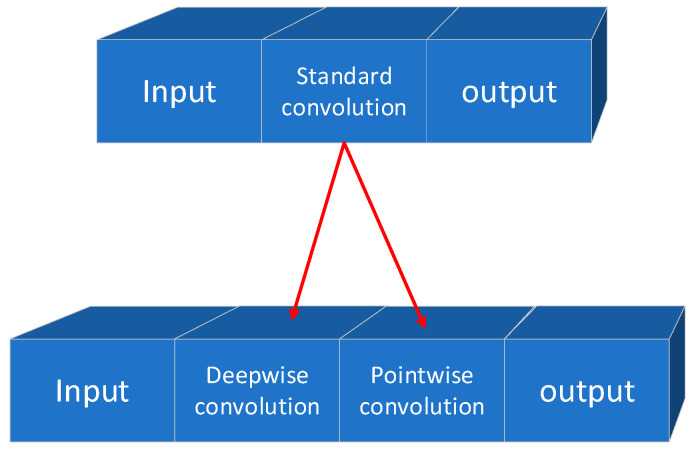
Depthwise separable convolution.

In 2017, Howard et al. [9] proposed MobileNet, a lightweight network for mobile and embedded vision applications. This network introduced two global hyperparameters, α (Width Multiplier) and ρ (Resolution Multiplier), that can be balanced in terms of latency and accuracy. The core components of MobileNet include depthwise separable convolution, width multiplier, and resolution multiplier. The standard convolution is decomposed into a pointwise convolution and a depthwise convolution, where the depth convolution is connected after the input channel, and the pointwise convolution is connected between the depth convolution and the output. This structure is illustrated in Figure 2.

**Figure 3:**
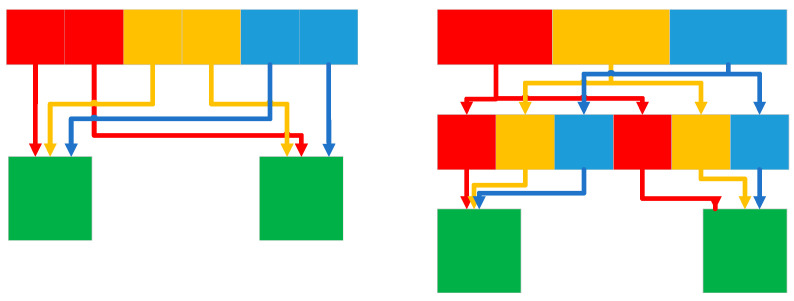
Shuffle group convolution operation.

Depthwise separable convolution separates the filtering and merging functions in standard convolution, using one layer for filtering and another layer for merging. This decomposition method can significantly reduce computational effort and model size. In MobileNet, when targeting specific applications that require smaller models and lower computational costs, the depth and point convolutions are computed as separate network layers, resulting in a total of 28 layers. In 2018, Zhang et al. [26] proposed ShuffleNet, a lightweight network for devices with computational constraints. The network architecture employs pointwise group convolution and channel shuffle techniques to significantly reduce computational costs while maintaining accuracy. Based on the efficient depth-separable convolution or group convolution of Xception [27] and ResNeXt [28], ShuffleNet considers 1 × 1 group convolution. The feature map generated for each group in the previous layer is divided into separate channels for several subgroups in each group to provide different subgroups for each group in the next layer. The Shuffle operation is shown in Figure 3.

Reference [29] proposed the once-for-all network (OFA), which can reduce training costs by selecting dedicated subnetworks without additional training. Additionally, the authors proposed a new progressive shrinkage algorithm for training OFA, which is a generalized pruning method. Experimental results showed that this method outperformed state-of-the-art NAS methods at the time and effectively reduced energy consumption. In another work [30], the network structure of Yolov5 was redesigned by embedding three network modules: CBAM, C3Ghost, and Ghost. The CBAM module was used to enhance the feature extraction capability, while C3Ghost and Ghost modules were used to reduce the number of parameters and floating-point operations. The MS COCO and PASCAL VOC datasets were used for experiments, and the results show that the new network structure had a slightly decreased average detection time, as well as a 50.61% reduction in floating-point operations and a 47.88% reduction in model parameters compared to the original network structure.

MobileNet has two more hyperparameters than SqueezeNet, which makes the application more flexible and able to be adjusted according to the actual application for computational cost and latency. ShuffleNet can generate more feature maps than SqueezeNet, but its deep convolution operation can only be performed on the bottleneck feature map, which leads to some difficulties in deployment on low-power devices. Additionally, most network structures are designed to adjust convolutional operations by replacing complex kernels with simpler ones to reduce computational complexity and the number of model parameters, while maintaining accuracy and reducing energy consumption. However, some network structures may have poor generalization ability and are only applicable in specific scenarios.

#### 3.1.2. Quantization

Quantization is the compression of floating-point data bits in neural network parameters to reduce model complexity and size by reducing the number of bits used by floating-point numbers, while maintaining model accuracy as much as possible.

In reference [31], BRECQ, a framework for Post-training Quantization (PTQ), was proposed for the first time to limit the bit-width range of the post-training quantization task to INT2. The authors conducted a comprehensive theoretical study of second-order errors and found that the framework was able to balance cross-layer dependence and generalization errors. They also used approximate inter-layer and intra-layer sensitivity, incorporating hybrid precision techniques. Experimental results show that post-training quantization can obtain a model with similar precision to ResNet and MobileNetV2 with only four bits using the Quantization-Aware Training (QAT) method without additional conditions, and can obtain 240 times the production speed of the quantized model. In another work [32], the authors propose data-free quantization methods that do not require data, fine-tuning, or hyperparameter optimization. They suggest a method that uses the scale-equivalence property of the activation function to adjust the range of weights in a network and corrects the errors introduced in the quantization process. Experiments show that the data-free quantization method approaches the original model’s accuracy and is even comparable to more sophisticated training-based methods. The authors of reference [33] propose a mechanism for weight-rounding for post-training quantization, AdaRound, which does not require fine-tuning of the network and can cope with data and task loss by using only a small amount of unlabeled data. Experiments show that this mechanism maintains accuracy loss within 1% by quantizing the weights of ResNet-18 and ResNet-50 to 4 bits. Reference [34] describes the “deep compression” method, a comprehensive approach that is a three-stage pipeline of pruning, training quantization, and Huffman (Huffman) coding running together. This approach can reduce the storage requirements of neural networks by a factor of 35 to 49 without compromising accuracy. The principle of the method is to first learn the significant connections as the basis for pruning the network, then quantize the weights to achieve weight sharing, and finally apply Huffman coding. Before Huffman coding, the authors retrained the network to fine-tune the remaining connections and quantized centroids, and reduced by nine to thirteen times the connections through pruning. Quantization reduced the connection bitwidth from 32 bits to 5 bits. Experimental results show that the deep compression approach reduces the storage space required for AlexNet by a factor of 35 from 240 mb to 6.9 mb on the AlexNet dataset with no loss of accuracy, and achieved 3 to 4 times the layered acceleration and 3 to 7 times the energy efficiency on CPU, GPU, and mobile GPU benchmarks. Finally, the authors of reference [35] propose the Efficient Inference Engine (EIE), which can be deployed to SRAM (Static Random-Access Memory) platforms. This engine utilizes the sparsity of activation functions and weights, and the technique of weight sharing and quantization. The EIE can save 120 times the energy, respectively, 10 times the energy by using sparsity, 8 times the energy by weight assignment, and 3 times the energy by skipping zero activation functions using ReLU. Unlike the large deep neural networks trained by the “deep compression” method, the EIE is suitable for on-chip DRAM (Dynamic Random-Access Memory).

Despite the various quantization methods mentioned above, there is still a need for better quantization methods that can achieve higher compression rates with lower accuracy degradation. Additionally, it is crucial to consider the energy consumption of the compressed network model to make it suitable for use in resource-constrained devices such as embedded devices.

#### 3.1.3. Pruning

Pruning is a method used to reduce redundant data in the neural network by determining the importance of each unit and removing unimportant parts.

One pruning method proposed in the literature [36] consists of three steps: first, train the network to learn the important connections; second, prune the unimportant connections; and finally retrain the network to adjust the weights of the remaining connections. Experimental results have shown that reducing the number of parameters by a factor of nine in the AlexNet network structure does not have a significant impact on performance. Another weight pruning method, ProbMask, was proposed in reference [37]. It measures the importance of weights by the probability of global criteria in all network layers and features automatic learning by setting constraints. Experimental results show that the ProbMask method can improve top-1 accuracy by about 10% compared with existing methods. Reference [38] proposes the ManiDp method, which maximizes the dynamic mining and pruning of redundant filters by embedding the manifold information of all instances into the pruned network space. The method achieves the dynamic removal of redundant filters, and experimental results show that it can reduce the number of floating-point operations by 55.3% while decreasing the top-1 accuracy by only 0.57% when applied on ResNet-34. A new channel exploration method, CHEX, is proposed in the literature [39] to solve the problem that traditional pruning methods require fully pre-trained large models and are limited by the pruning process. CHEX repeatedly prunes and regrows the channels during the training process, reducing the risk of prematurely pruning important channels. Experimental results show that a top-1 accuracy of 76% can be obtained using the CHEX compressed ResNet-50 model on the ImageNet dataset, reducing the Flops (floating point operations per second) to only 25% of the original ResNet-50 model. Finally, reference [40] proposes a channel pruning method that uses a random search method to determine the channel model of the pruned network structure. Experimental results show that the performance of the models obtained with different network structures and datasets is close under the random pruning scheme. However, the number of parameters has a great impact on the accuracy of the constructed networks, and the more parameters the lower the error rate of the pruned network after a certain amount of computation.

In conclusion, current pruning methods include weight pruning, channel pruning, and neuron pruning, each with its advantages and disadvantages. The criteria for determining the importance of the unit can impact the accuracy. The use of constraint learning methods can improve it, although the implementation is complex. The random search method of pruning is simple to implement, but the network model’s performance is limited compared to other methods that improve it. Therefore, each method has its advantages and needs to be selected in conjunction with the actual application scenario, and there is no single method that can synthesize the complexity and model compression efficiency.

### 3.2. Binary Neural Networks and Optimization Techniques

Convolutional neural networks consist of multiple network layers and millions of parameters. Due to their large size, it is challenging to deploy them directly on embedded devices that have high hardware requirements.

**Figure 4:**
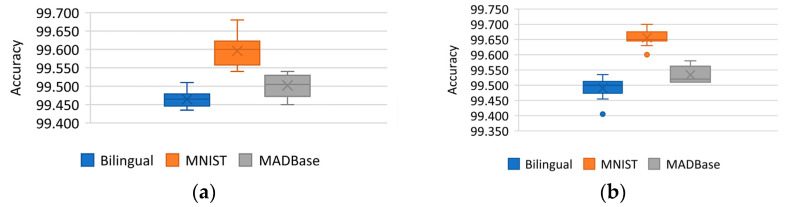
Reference [45] (**a**) CS-LBCNN experiment result; (**b**) TCS-LBCNN experiment result.

To address this issue, a binarization method was proposed to simplify the network parameters [41]. This method quantifies the weights and activation values into one fixed-point parameter, leading to memory savings and reduced inference time. However, the binarization network results in severe information loss. The direction of binarization network research is towards reducing information loss, reducing errors, and preserving model accuracy. The authors of reference [42], Courbariaux et al., proposed the concept of binarized neural networks and used a randomized binarization method during the training forward propagation. During the backward propagation, a clip function was introduced to intercept the full precision weight to update the range, compressing the number of network model parameters to a great extent and preventing the real weights from growing too fast without affecting the binary weights. One year later, after the Binary network was proposed, reference [43] proposed Binary-Weight-Networks and XNOR-Networks. The filters of binary-weighted networks approximate binary values, and XNOR-Networks filters and convolutional layer inputs are binary, which accelerates the speed of convolutional operation and saves 32 times the memory. The experiments using the ImageNet dataset show that the method improved the top-1 accuracy by 16% compared to other network binarization methods used at that time. Reference [44] presents the first hash method training binary method and experiments on CIFAR-10, CIFAR-100 with ImageNet dataset. The main work of the authors was to convert the training binary network into a hash problem, multiply the binary code by a scaling factor to reduce the loss caused by using the hash method, and propose alternate optimization methods to iteratively update the binary code and the scaling factor. Experimental results show a 3.0% improvement in accuracy for the ImageNet classification task compared to the best binarization network at that time. Reference [45] proposes Center-Symmetric Local Binary Convolutional Neural Networks (CS-LBCNN) for handwritten character recognition to address the problem that local binary networks are affected by randomly assigned local binary convolutional weights. The authors also propose an improvement—Threshold Center-Symmetric Local Binary Convolutional Neural Networks (TCS-LBCNN). The experiments were compared in CS-LBCNN and TCS-LBCNN using bilingual, MNIST, and MADBase datasets, and the average accuracies obtained in CS-LBCNN were 99.465%, 99.596%, and 99.502%, respectively. The final average accuracies achieved in TCS-LBCNN were 99.491%, 99.656%, and 99.534%. Experimental result showing in Figure 4. The authors also compared with today’s advanced techniques and were able to achieve a small number of improvements in performance and accuracy.

In reference [46], the AdaBin method was proposed to adaptively obtain the optimal binary set {b1, b2} for each layer of weights and activations. The method defined a new binary quantization function using the center position and distance of 1-bit values. An equalization method was proposed for the weights to minimize their Kullback–Leibler scatter, and a gradient-based optimization method was introduced to obtain the activation parameters. Experimental results showed that a top-1 precision of 66.4% could be obtained using the ResNet-18 architecture on the ImageNet dataset, and 69.4mAp (mean Average Precision, which is a synthesis of both Precision and Recall metrics) was obtained using SSD300 on the PASCAL VOC.

Binarization techniques are currently available to significantly reduce the size and complexity of models, enabling complex neural networks to be deployed on resource-constrained devices. However, accuracy loss remains a serious issue. To address this, researchers have explored using other values besides the traditional {−1, 1} for weights or activation values, increasing the complexity of the network to improve accuracy. Alternatively, adaptive binarization methods may be the future direction. These methods can dynamically adjust the range of weights and activation values based on the situation, providing a more flexible and accurate approach.

### 3.3. CPU/GPU Acceleration Algorithm

In addition to optimizing network performance, hardware-based CPU and GPU algorithms for neural network acceleration can also be optimized in the field of computer science. The current CPU/GPU acceleration algorithms for neural networks are mainly divided into three categories: adjusting the task scheduling strategy, enhancing CPU-GPU parallel computing efficiency, and strengthening GPU utilization.

Reference [47] proposed the RedSync method based on the RGC (Residual Gradient Compression) compression algorithm, which can reduce end-to-end training time in multi-GPU systems. This method significantly accelerated the training speed of large-scale deep neural networks. Reference [48] proposed the RedSync method based on the residual gradient compression (RGC) compression algorithm, which can reduce the end-to-end training time in multi-GPU systems. This method significantly accelerates the training speed of large-scale deep neural networks. In reference [9], the authors propose Troodon, a load-balanced scheduling heuristic suitable for CPUs and GPUs. The main idea of the algorithm is to organize all jobs into job pools according to device suitability, rank the jobs in the job pools according to the predicted speedup rate, and achieve load-balanced scheduling by considering the processing requirements of the jobs and the computational capacity of the devices. This is calculated by computing the compute shares of each device that are related to the available workload to be scheduled. The authors’ experiments were conducted on two CPU-GPU heterogeneous systems, and Troodon’s final processing time was reduced by 78%, 65%, and 41%, respectively, compared to the other three algorithms in the literature (DS, ISG, and AA). In reference [49], the authors propose methods to execute a local respective-field-based Extreme Learning Machine (LU) on a GPU platform. The first method is a new chunked logical unit decomposition algorithm that overcomes the global memory size limitation. The second method is used to accelerate the chunked LU decomposition efficient chunking algorithm Cholesky decomposition algorithm. Finally, the authors propose a heterogeneous CPU-GPU parallel algorithm that can make full use of GPU node resources. The experimental results showed that the chunking Cholesky decomposition algorithm was two times faster compared to the LU decomposition algorithm, while the heterogeneous blocking CPU-GPU acceleration algorithm improved the performance by 5% to 10% compared to the Cholesky algorithm. The authors of reference [50] propose Hybrid Parallel DESPOT (HyP-DESPOT), a massively parallel online planning algorithm based on the DESPOT algorithm that integrates CPU and GPU parallelism in a multilevel scheme for robotic environments. The DESPOT algorithm has three key steps—1. Forward Search, 2. Leaf node initialization, and 3. Backup—with the two steps of forward search and backup being irregular. The experimental results show that the execution speed of the HyP-DESPOT algorithm is significantly faster than that of DESPOT. In addition to CPU and GPU optimization for neural networks, specialized components can also be designed for neural network acceleration, such as the hardware-efficient vector convolutional neural network accelerator proposed in reference [51], which uses a 3 × 3 filter to optimize the shrink array. Here, a one-dimensional broadcast data stream is used to generate partial sums, thus compressing the computational overhead. The experiments in this paper mainly measure the improved neural network energy consumption and hardware utilization of the convolutional neural network accelerator applied to VGG-16, ResNet-34, GoogleNet, and MobileNet. The results show that the power consumption of the network loaded with the acceleration was reduced, and its hardware utilization reached 99%, 97%, 93.7%, and 94%. Reference [52] proposes kernel merging methods to improve GPU occupancy and also proposes a machine learning-based GPU sharing mechanism to select kernel pairs in the OpenCL framework. The approach in this work first selects a suitable architecture for the task and then merges GPU cores to improve resource utilization. Experimental results show that the model built using this method achieves 0.91 and 0.98 in F-measure.

The current CPU/GPU algorithms for neural network acceleration have improved support for embedded devices, and research is focused on optimizing their operational efficiency. However, the issue of energy consumption has been largely overlooked. It is important to consider the impact of these algorithms on energy consumption in future research.

### 3.4. Summary

This section introduces three key technologies: Model Compression, Binary networks, and CPU/GPU acceleration algorithms, respectively. These three technologies will determine whether embedded AI can deploy more complex and efficient models and algorithms in the future. Furthermore, it is important to consider how the network structure and hardware algorithm support can be better implemented in the future to facilitate the integration with IoT edge computing. Table 2 provides the characteristics of each work covered in this section.

## 4. Application Modes of Embedded Artificial Intelligence

The application of embedded AI can be simply divided into three approaches: deploying trained models and weights and other data to embedded devices, training on embedded devices, and training partially on embedded devices and partially on other devices. The first two approaches have their characteristics. Post-training deployment is simpler, but it requires more computational resources in inference. Embedded device training can avoid the bottleneck of data transmission and computational resources, but training on embedded devices requires more computational resources and time compared to post-training deployment. The third approach is currently less studied, but task offloading in edge computing is more compatible with this direction. Table 3 shows the literature reviewed in this chapter.

### 4.1. Post-Training Deployment

The authors of reference [53] utilized a wearable smart log patch to monitor and compare health data, such as blood pressure, temperature, and electrocardiogram readings. The wearable IoT smart log patch had a hardware specific chip size of 5 mm, operated at 0.9 V with a 120 Mhz clock speed, and had built-in Wi-Fi and Bluetooth, 14 I/O pins, and 12 sensors. The formula for accuracy in this work was as follows.

(3) $$Accuracy=\frac{F(TruePostive)+F(TrueNegative)}{F(TruePostive)+F(TrueNegative)+F(FalsePostive)+F(FalseNegative)}$$

The article proposes an EC-BDLN algorithm, which reduces data faults during the transportation of information from the input layer to the output layer, resulting in high throughput. Additionally, when the network detects a data fault, it can transport data from the source to the destination address using minimal energy consumption. The algorithm also introduces a Gaussian factor that helps to maintain unwanted switching activities in the network and improve the prediction accuracy. In the experiments, the proposed algorithm outperforms other methods in terms of efficiency and has lower error rates. Reference [54] describes a wearable fall monitoring system that uses a wearable motion sensor with three axial devices, including a compass/magnetometer, gyroscope, and accelerometer. The proposed artificial intelligence IOT-CPS (Cyber-Physical Systems) diagnoses patient disease accuracy, recall, F-Measure, and execution time, and shows significant improvement over existing algorithms. Reference [55] is about deploying machine-learning capabilities on a smartphone mobile platform for target recognition. The proposed target detection system is based on two platforms: OpenCV and Tensorflow. The UAV configuration can transmit video at 720p/1080p with low latency and high quality. The controller is wirelessly connected to the UAV and the Android device, and these three components are synchronized. To deploy Yolov3 on an Android mobile device, the authors used Keras to process the network configuration file and the weight file. This generated an intermediate file with the extension h5, as well as a help file that defines the neural network in JSON format. In their experiments, the authors used SSD, YOLOv3, and Tiny-YOLOv3 deployed on two platforms to evaluate the accuracy, speed, RAM, energy consumption, and temperature. The authors found that OpenCV outperforms TensorFlow when performing image preprocessing on mobile devices. However, TensorFlow performs better when the tracking process is running, and it also has the highest neural network execution speed. TensorFlow significantly outperforms other platforms, achieving a higher number of frames per second. In reference [56], the authors explored artificial intelligence for basic hand position recognition in Chinese classical dance, using TensorFlow on NVIDIA Jeston series embedded AI development boards. They extracted the coordinates of human joint point positions in the video and calculated the similarity between imitators’ action poses and standard action samples using cosine similarity algorithms to achieve recognition and evaluation of academic poses in Chinese classical dance. Finally, reference [57] investigates image segmentation, feature extraction, and motion recognition methods based on embedded systems and artificial intelligence platforms for taekwondo technical action processes and motion effects. The authors optimized their algorithm, involving improved background subtraction for image segmentation and the use of principal component analysis to construct feature vectors to reduce the number of dimensions of the quantitative description of features. They chose a microcontroller containing max1300 chip and max9632 chip for deployment and achieved a desirable recognition rate for 10 actions in the video database. However, this work did not deploy the algorithm on a real-time system, only mentioning the possibility.

### 4.2. Training on Embedded Devices

In [4], the authors propose a zero-activation-skipping convolutional accelerator (ZASCA), which avoids the non-contributing multiplication of zero-valued activations. The main submodules are convolutional units (CEs), pipelines, and encoding. In the experiments, the authors denote ZASCA, which performs convolution on dense activation, as ZASCAD, and ZASCAS, which performs convolution on sparse activation, as ZASCAS. They compare ZASCAD and ZASCAS with Eyeriss, EEPS, and DSIP using AlexNet, VGG-16, ResNet-18, and ResNet-50 datasets. Eyeriss is tested on AlexNet and VGG-16, and EEPS and DSIP are tested on AlexNet only. The results show that when running AlexNet and VGG-16, ZASCAD runs 1.4 times and 3.1 times faster than Eyeriss, respectively, and ZASCAS performs AlexNet convolution 1.5 times faster than EEPS. Finally, ZASCAD and ZASCAS perform 2.7 times and 4 times better than DSIP at runtime, respectively. The authors also provide an energy consumption analysis in the paper, which yields the result that ZASCAS performs ResNet-50 convolution to obtain the highest energy efficiency. In [58], the authors used computational intelligence techniques for image analysis to study predictive computational intelligence techniques for the nitrogen status evaluation of wheat crops using Hue–Saturation–Intensity color normalization, genetic algorithm (GA), and artificial neural network (ANN) to predict the crop accuracy status and classify it. They propose an accurate crop yield prediction model that combines ANN and GA to predict crop yield by first extracting low-level color, morphology, and texture features, then further processing using GA, and finally optimizing the features for transmission to the ANN for crop prediction. The experiments in this work were conducted on an Intel Core i5 CPU using a dataset of 18,200 images of wheat crops, divided into four categories based on the age of the wheat, with 5460 images for testing and 12,740 images for the training and implementation of the neural network. The experimental results verified an accuracy of 97.75%, with a minimum error of 0.22 and a loss value reduction of 0.28 compared to other contemporary methods. After comparative experimental analysis, the model provided an effective and reliable approach to the crop yield prediction problem due to the optimization of the training parameters and the low hardware requirements of the method using ANN with GA. In [59], the authors proposed an energy-efficient thin and deep convolutional neural network architecture dedicated to traffic sign recognition, which contained four convolutional layers, two overlapping maximum pooling layers, and one fully connected hidden layer. Using overlapping maximum pooling layers instead of non-overlapping maximum pooling layers can solve the problem of overfitting and compress the image before processing. The authors used an Intel Core i7 CPU for training, and the datasets were German traffic sign recognition benchmark and Belgian traffic sign classification datasets. The final experimental results showed that the proposed architecture outperforms state-of-the-art traffic sign methods and reduces the system energy consumption due to the lower computational effort of the method used in the literature. Since the computational cost is linearly equivalent to the energy consumption, the method achieves reduced energy consumption and complexity while maintaining the accuracy of other contemporary methods. However, the network structure was designed exclusively for traffic sign recognition and lacked generalization.

### 4.3. Partial Training

In reference [5], the authors proposed incorporating deep learning into the edge computing environment to enhance learning performance while reducing network traffic. By utilizing deep learning, edge computing can extract important information and reduce the communication overhead transmitted from edge devices to the cloud server. Additionally, some of the learning layers can be implemented at the edge during model training, and only the reduced intermediate data are transferred to the centralized cloud server. Experimental results demonstrate that this approach outperforms other methods in optimizing IoT deep learning.

### 4.4. Summary

Currently, most embedded AI applications are deployed on embedded devices after being trained elsewhere. However, research on on-device training and the execution of tasks is relatively scarce. These two deployment modes have different requirements and are more difficult to match. On-device training can lead to better generalization, but also requires higher hardware capabilities. In the future, the development of hardware accelerators will likely promote the development of both deployment modes.

## 5. The Outlook of Embedded Artificial Intelligence

In the Industry 4.0 environment, the digitalization process of the manufacturing industry relies on embedded intelligence technology. To achieve this, more complex and intelligent artificial intelligence algorithms and models need to be deployed to resource-constrained embedded devices. Embedded intelligence will play an important role in the digital transformation of the manufacturing industry. The following are some considerations for embedded artificial intelligence technology:

1. Efficient algorithms and lightweight models: In the current society, most workers need to frequently switch between different work scenarios. This results in higher requirements for device portability, including weight, volume, energy consumption, and other factors. To ensure the portability of the devices, the development of intelligent devices requires the study of more efficient algorithms and lightweight network models while maintaining model accuracy and reducing network model complexity.
2. Hardware acceleration methods: In addition to optimization in algorithms and models, optimization can also be achieved at the hardware level. The current research on hardware acceleration methods is limited to a single architecture of the neural network accelerator. Applying a hardware neural network accelerator to multiple platforms or using multiple hardware devices in combination may become a solution to the problem in the future.
3. Deployment optimization: Embedded AI deployment can be divided into post-training deployment, training on embedded devices, and part of the training task on embedded devices. Current post-training deployment has a high demand for training speed on other platforms, which can be met by improving the model training speed. The need for training on embedded devices is consistent with the first point of this subsection, requiring more efficient algorithms and lighter network models to reduce the difficulty of model training on embedded devices. For tasks completed on embedded devices, consideration of post-training models for integration is required to ensure model integrity.
4. Compatibility: According to reference [60], the current embedded intelligence in the industry still faces problems. For example, in legacy automation systems, some dedicated functions lack interoperability with the current automation system due to various reasons. At the same time, there is no standard method to manage the edge computing nodes and data collection. Additionally, utilizing the large amount of data generated by the edge computing and industrial cloud working together in machine learning remains an issue.

## 6. Conclusions

This paper presents the current state of development in embedded AI from three perspectives: hardware acceleration methods, key technologies, and application models. Currently, embedded intelligence has established a foundation that includes deployment platforms for AI support, such as designing hardware accelerators for neural networks, optimizing network models, which includes network structure design, quantization, pruning, and binarization methods, and improving underlying hardware algorithms. All of the above technologies contribute to the deployment of AI on resource-constrained devices, but further development is needed in the following areas: efficient algorithms and lightweight models, the optimization of hardware acceleration methods, and the optimization of deployment methods and compatibility.

## Figures and Tables

**Table 1:** Literature on hardware accelerators.

Classification	Reference	Proposed Method	Advantage	
FPGA	[12]	Pre-implemented CNN Accelerator	Lower resource and high energy efficiency	Flexibility and Scalability
	[13]	ASimOV framework	Lower memory high performance	
	[14]	Compare between ANN and SNN	SNN is better than ANN in energy efficiency and inference speed	
	[15]	fpgaConvNet Accelerator	Higher energy efficiency and raw performance	
	[16]	Synchronous dataflow	Higher performance and performance density	
ASIC	[17]	YodaNN CNN Accelerator	Higher performance and energy efficiency	Performance and Energy Efficiency
	[18]	UCNN Accelerator	Lower computation cost	
	[19]	MSICs	Higher energy efficiency	
GPU	[20]	CRYPTGPU	Faster than CPU and more private	Parallel Computing Capabilities
	[21]	ParSecureML	Faster than other SecureML frameworks	
Other	[22]	NPU application system	More efficient and lower energy consumption	Customization
	[23]	NVIDIA Jetson	Powerful parallel computation capability	

**Table 2:** Literature covering key technologies.

Classification	Reference	Proposed Method	Advantage
Model Compression			
Network Design	[25]	SqueezeNet	Fewer parameters
	[9]	MobileNet	Compatible Resource-scarce embedded devices
	[31]	ShuffleNet	
	[29]	Once-for-all network	Lower energy consumption
	[41]	Improvement of Yolov5	Faster detection speed
Quant	[31]	BRECQ	Faster production
	[32]	Data-Free Quantization	Less precision loss
	[33]	AdaRound	Less precision loss
Quant	[11]	DeepCompression	Fewer storage requirements with no loss of precision
	[35]	Efficient Inference Engine	Lower energy consumption
Prune	[25]	Important Connection Pruning	Fewer parameters
	[37]	ProbMask	Higher precision
	[38]	ManiDp	Fewer flops
	[39]	CHEX	Fewer flops and less precision loss
	[15]	Channel pruning	Less loss of performance
Binary Neural Network			
	[42]	Binary Neural Network	Significant reduction in the number of parameters
	[43]	XNOR-Network	Less memory consumption
	[44]	From hashing to CNM	Improvement of accuracy
	[45]	CS-LBCNN and TCS-LBCNN	Higher precision
	[46]	AdaBin	Less loss of precision
CPU/GPU Acceleration			
	[47]	RedSync	Faster training speed
	[9]	Troodon	Less processing time
	[34]	Local respective field-based Extreme Learning Machine	Higher performance and faster decomposition speed
	[50]	HyP-DESPOT	Faster execution Speed
	[51]	Hardware efficient vector-wise accelerator	Less energy consumption and higher hardware utilization
	[52]	GPU-kernel fusion model	Higher F-measure

**Table 3:** Literature on application modes.

Classification	Reference
Post-Training Deployment	[31,53,54,55,56,57]
Training on Embedded Devices	[4,58,59]
Partial Training	[31]

## Data Availability

The data that support the findings of this study are available from the corresponding author upon reasonable request.

## References

[1] Ang K.L.-M., Seng J.K.P. (2021). Embedded Intelligence: Platform Technologies, Device Analytics, and Smart City Applications. IEEE Internet Things J..

[2] Dick R.P., Shang L., Wolf M., Yang S.-W. (2019). Embedded Intelligence in the Internet-of-Things. IEEE Des. Test.

[3] Guo B., Zhang D., Yu Z., Liang Y., Wang Z., Zhou X. (2012). From the internet of things to embedded intelligence. World Wide Web.

[4] Ardakani A., Condo C., Gross W.J. (2019). Fast and Efficient Convolutional Accelerator for Edge Computing. IEEE Trans. Comput..

[5] Li H., Ota K., Dong M. (2018). Learning IoT in Edge: Deep Learning for the Internet of Things with Edge Computing. IEEE Netw..

[6] Manavalan E., Jayakrishna K. (2018). A review of Internet of Things (IoT) embedded sustainable supply chain for industry 4.0 requirements. Comput. Ind. Eng..

[7] Xu D., Li T., Li Y., Su X., Tarkoma S., Jiang T., Crowcroft J., Hui P. (2021). Edge Intelligence: Empowering Intelligence to the Edge of Network. Proc. IEEE.

[8] Poniszewska-Maranda A., Kaczmarek D., Kryvinska N., Xhafa F. (2018). Studying usability of AI in the IoT systems/paradigm through embedding NN techniques into mobile smart service system. Computing.

[9] Howard A.G., Zhu M., Chen B., Kalenichenko D., Wang W., Weyand T., Andreetto M., Adam H.J. (2017). Mobilenets: Efficient convolutional neural networks for mobile vision applications. arXiv.

[10] Deng B.L., Li G., Han S., Shi L., Xie Y. (2020). Model Compression and Hardware Acceleration for Neural Networks: A Comprehensive Survey. Proc. IEEE.

[11] Krishnamoorthi R.J. (2018). Quantizing deep convolutional networks for efficient inference: A whitepaper. arXiv.

[12] Kwadjo D.T., Tchinda E.N., Mbongue J.M., Bobda C. (2022). Towards a component-based acceleration of convolutional neural networks on FPGAs. J. Parallel Distrib. Comput..

[13] Hwang D.H., Han C.Y., Oh H.W., Lee S.E. (2021). ASimOV: A Framework for Simulation and Optimization of an Embedded AI Accelerator. Micromachines.

[14] Li Z., Lemaire E., Abderrahmane N., Bilavarn S., Miramond B. (2022). Efficiency analysis of artificial vs. Spiking Neural Networks on FPGAs. J. Syst. Arch..

[15] Venieris S.I., Bouganis C.S. (2017). fpgaConvNet: A toolflow for mapping diverse convolutional neural networks on embedded FPGAs. arXiv.

[16] Venieris S.I., Bouganis C.-S. (2018). fpgaConvNet: Mapping Regular and Irregular Convolutional Neural Networks on FPGAs. IEEE Trans. Neural Networks Learn. Syst..

[17] Andri R., Cavigelli L., Rossi D., Benini L. (2017). YodaNN: An Architecture for Ultralow Power Binary-Weight CNN Acceleration. IEEE Trans. Comput. Des. Integr. Circuits Syst..

[18] Hegde K., Yu J., Agrawal R., Yan M., Pellauer M., Fletcher C. UCNN: Exploiting Computational Reuse in Deep Neural Networks via Weight Repetition. Proceedings of the 2018 ACM/IEEE 45th Annual International Symposium on Computer Architecture (ISCA).

[19] Shin D., Yoo H.-J. (2019). The Heterogeneous Deep Neural Network Processor With a Non-von Neumann Architecture. Proc. IEEE.

[20] Wang M., Yang T., Flechas M.A., Harris P., Hawks B., Holzman B., Knoepfel K., Krupa J., Pedro K., Tran N. (2021). GPU-Accelerated Machine Learning Inference as a Service for Computing in Neutrino Experiments. Front. Big Data.

[21] Zhang F., Chen Z., Zhang C., Zhou A.C., Zhai J., Du X. (2021). An Efficient Parallel Secure Machine Learning Framework on GPUs. IEEE Trans. Parallel Distrib. Syst..

[22] Kang M., Lee Y., Park M. (2020). Energy Efficiency of Machine Learning in Embedded Systems Using Neuromorphic Hardware. Electronics.

[23] Mittal S. (2019). A Survey on optimized implementation of deep learning models on the NVIDIA Jetson platform. J. Syst. Arch..

[24] Liu X., Ounifi H.-A., Gherbi A., Li W., Cheriet M. (2019). A hybrid GPU-FPGA based design methodology for enhancing machine learning applications performance. J. Ambient. Intell. Humaniz. Comput..

[25] Iandola F.N., Han S., Moskewicz M.W., Ashraf K., Dally W.J., Keutzer K.J. (2016). SqueezeNet: AlexNet-level accuracy with 50× fewer parameters and<0.5 MB model size. arXiv.

[26] Zhang X., Zhou X., Lin M., Sun J. (2017). ShuffleNet: An Extremely Efficient Convolutional Neural Network for Mobile Devices. arXiv.

[27] Chollet F. Xception: Deep learning with depthwise separable convolutions. Proceedings of the IEEE Conference on Computer Vision and Pattern Recognition 2017.

[28] Xie S., Girshick R., Dollár P., Tu Z., He K. In Aggregated residual transformations for deep neural networks. Proceedings of the IEEE Conference on Computer Vision and Pattern Recognition 2017.

[29] Cai H., Gan C., Wang T., Zhang Z., Han S. (2019). Once-for-all: Train one network and specialize it for efficient deployment. arXiv.

[30] Dong X., Yan S., Duan C. (2022). A lightweight vehicles detection network model based on YOLOv5. Eng. Appl. Artif. Intell..

[31] Li Y., Gong R., Tan X., Yang Y., Hu P., Zhang Q., Yu F., Wang W., Gu S. (2021). Brecq: Pushing the limit of post-training quantization by block reconstruction. arXiv.

[32] Nagel M., Van Baalen M., Blankevoort T., Welling M. Data-Free Quantization Through Weight Equalization and Bias Correction. Proceedings of the IEEE/CVF International Conference on Computer Vision 2019.

[33] Nagel M., Amjad R.A., Van Baalen M., Louizos C., Blankevoort T. Up or down? adaptive rounding for post-training quantization. Proceedings of the International Conference on Machine Learning 2020.

[34] Han S., Mao H., Dally W.J. (2015). Deep compression: Compressing deep neural networks with pruning, trained quantization and huffman coding. arXiv.

[35] Han S., Liu X., Mao H., Pu J., Pedram A., Horowitz M.A., Dally W.J. (2016). EIE: Efficient inference engine on compressed deep neural network. ACM SIGARCH Comput. Archit. News.

[36] Han S., Pool J., Tran J., Dally W. (2015). Learning both weights and connections for efficient neural network. Adv. Neural Inf. Process. Syst..

[37] Zhou X., Zhang W., Xu H., Zhang T. Effective sparsification of neural networks with global sparsity constraint. Proceedings of the IEEE/CVF Conference on Computer Vision and Pattern Recognition, 2021.

[38] Tang Y., Wang Y., Xu Y., Deng Y., Xu C., Tao D., Xu C. Manifold regularized dynamic network pruning. Proceedings of the IEEE/CVF Conference on Computer Vision and Pattern Recognition, 2021.

[39] Hou Z., Qin M., Sun F., Ma X., Yuan K., Xu Y., Chen Y.-K., Jin R., Xie Y., Kung S.-Y. Chex: Channel exploration for CNN model compression. Proceedings of the IEEE/CVF Conference on Computer Vision and Pattern Recognition, 2022.

[40] Li Y., Adamczewski K., Li W., Gu S., Timofte R., Van Gool L. Revisiting random channel pruning for neural network compression. Proceedings of the IEEE/CVF Conference on Computer Vision and Pattern Recognition, 2022.

[41] Courbariaux M., Hubara I., Soudry D., El-Yaniv R., Bengio Y. (2016). Binarized neural networks: Training deep neural networks with weights and activations constrained to+ 1 or-1. arXiv.

[42] Courbariaux M., Bengio Y., David J.P. (2015). Binaryconnect: Training deep neural networks with binary weights during propagations. Adv. Neural Inf. Process. Syst..

[43] Rastegari M., Ordonez V., Redmon J., Farhadi A. (2016). In Xnor-net: Imagenet classification using binary convolutional neural networks. Proceedings of the Computer Vision–ECCV 2016: 14th European Conference.

[44] Hu Q., Wang P., Cheng J. (2018). From Hashing to CNNs: Training Binary Weight Networks via Hashing. Proc. Conf. AAAI Artif. Intell..

[45] Al-Wajih E., Ghazali R. (2023). Threshold center-symmetric local binary convolutional neural networks for bilingual handwritten digit recognition. Knowledge-Based Syst..

[46] Tu Z., Chen X., Ren P., Wang Y. (2022). Adabin: Improving Binary Neural Networks with Adaptive Binary Sets, Proceedings of the Computer Vision–ECCV 2022: 17th European Conference, Tel Aviv, Israel, 23–27 October 2022.

[47] Fang J., Fu H., Yang G., Hsieh C.-J. (2019). RedSync: Reducing synchronization bandwidth for distributed deep learning training system. J. Parallel Distrib. Comput..

[48] Khalid Y.N., Aleem M., Ahmed U., Islam M.A., Iqbal M.A. (2019). Troodon: A machine-learning based load-balancing application scheduler for CPU–GPU system. J. Parallel Distrib. Comput..

[49] Li S., Niu X., Dou Y., Lv Q., Wang Y. (2017). Heterogeneous blocked CPU-GPU accelerate scheme for large scale extreme learning machine. Neurocomputing.

[50] Cai P., Luo Y., Hsu D., Lee W.S. (2021). HyP-DESPOT: A hybrid parallel algorithm for online planning under uncertainty. Int. J. Robot. Res..

[51] Chang K.-W., Chang T.-S. (2019). VWA: Hardware Efficient Vectorwise Accelerator for Convolutional Neural Network. IEEE Trans. Circuits Syst. I Regul. Pap..

[52] Ahmed U., Lin J.C.-W., Srivastava G. (2022). A ML-based resource utilization OpenCL GPU-kernel fusion model. Sustain. Comput. Inform. Syst..

[53] Manogaran G., Shakeel P.M., Fouad H., Nam Y., Baskar S., Chilamkurti N., Sundarasekar R. (2019). Wearable IoT Smart-Log Patch: An Edge Computing-Based Bayesian Deep Learning Network System for Multi Access Physical Monitoring System. Sensors.

[54] Ramasamy L.K., Khan F., Shah M., Prasad B.V.V.S., Iwendi C., Biamba C. (2022). Secure Smart Wearable Computing through Artificial Intelligence-Enabled Internet of Things and Cyber-Physical Systems for Health Monitoring. Sensors.

[55] Martinez-Alpiste I., Casaseca-de-la-Higuera P., Alcaraz-Calero J.M., Grecos C., Wang Q. (2020). Smartphone-based object recognition with embedded machine learning intelligence for unmanned aerial vehicles. J. Field Robot..

[56] Zhou Q., Wang J., Wu P., Qi Y. (2021). Application Development of Dance Pose Recognition Based on Embedded Artificial Intelligence Equipment. J. Physics Conf. Ser..

[57] Ma Q., Wang Y. (2021). RETRACTED ARTICLE: Application of embedded system and artificial intelligence platform in Taekwondo image feature recognition. J. Ambient. Intell. Humaniz. Comput..

[58] Sharma A., Georgi M., Tregubenko M., Tselykh A., Tselykh A.J.C., Engineering I. (2022). Enabling smart agriculture by implementing artificial intelligence and embedded sensing. Comput. Ind. Eng..

[59] Haque W.A., Arefin S., Shihavuddin A., Hasan M.A. (2020). DeepThin: A novel lightweight CNN architecture for traffic sign recognition without GPU requirements. Expert Syst. Appl..

[60] Dai W., Nishi H., Vyatkin V., Huang V., Shi Y., Guan X. (2019). Industrial Edge Computing: Enabling Embedded Intelligence. IEEE Ind. Electron. Mag..

